# The plasmatic and salivary levels of IL-1β, IL-18 and IL-6 are associated to emotional difference during stress in young male

**DOI:** 10.1038/s41598-018-21474-y

**Published:** 2018-02-14

**Authors:** I. La Fratta, R. Tatangelo, G. Campagna, A. Rizzuto, S. Franceschelli, A. Ferrone, A. Patruno, L. Speranza, M. A. De Lutiis, M. Felaco, A. Grilli, M. Pesce

**Affiliations:** Medicine and Health Science School, Via dei Vestini, 31-66100 Chieti, Italy

## Abstract

Saliva collection is considered a non-invasive method to detect inflammatory markers in response to emotional states within natural social contexts. Numerous studies have prompted an important role of cytokines in modulating distinct aspects of social and emotional behavior. The aim of this study was to investigate the reliability of plasma and saliva as investigative tools for measure some inflammatory marker levels (CRP, IL-1β, IL-18, and IL-6). At the same time, the relationships between these markers and emotional states in response to a socio-cognitive stress (Academic Exam, AE), were considered. It was demonstrated that the plasma and saliva concentrations of all immune-mediators analyzed were significantly related across the socio-cognitive stress. In addition, when there was a close correlation to AE, the anger state, the IL-1β, the IL-18 salivary and plasmatic concentrations were significantly higher, while they decreased during the AE. On the other hand, the anxiety state and the IL-6 levels significantly increased throughout the AE. The IL-1β and IL-6 were positively associated to the anger and the anxiety state, respectively. In conclusion, our data highlight that different immune markers are similarly detectable in plasma and saliva during socio-cognitive stress. Also, they could be related to different emotional responses.

## Introduction

Several studies examining the emotional states in relation to immune markers in serum or plasma have shown that the latter is still considered the best body fluid for several biomarkers that reflect systemic processes^1–4^. However, the opportunity to determine several immune markers in saliva has improved the possibility to study the relationship between immune mediators and emotional and behavioral changes^5,6^.

The diagnosis on the analysis of saliva has acquired great interest because saliva is a non-invasive method of collection and represents an alternative stress-free serum sample. In addition to being non-invasive, the assessment through the saliva allows data collection to take place in the participants’ ecological context (e.g., home, university), and can be repeated over time. Moreover, saliva collection has considerable economical and logistic advantages over venipuncture because it does not require immediate manipulations, access to specialized laboratory equipments and qualified personnel.

This substrate is usually used in dentistry and for studies in oral diseases^7^, and is a useful tool in determining hormonal and immunity markers in the area of psycho-physiology. For example, salivary Cortisol (C) sampling has been used as a measure of Hypothalamus Pituitary Adrenal (HPA) axis activity for some time, as there is a high correlation between salivary C levels and unbound free C levels in plasma and serum which remain high during the circadian cycle^8^.

Several studies have highlighted a key role of cytokines in modulating different aspects of social and emotional behavior. The levels of inflammatory cytokines have been positively linked to stressful experiences as well to certain emotions in studies that used the trait measures^9^ or to negative moods^10–12^. In accord, in clinical conditions characterized by a negative affective trait (i.e. depression and anxiety), the level of pro-inflammatory cytokines is higher^13–15^, highlighting the interplay between emotional experiences and immune responses.

The immune system is organized into anatomically discrete compartments to respond to environmental stimuli within different body tissues (e.g. blood, oral cavity, intestines and airways). Saliva is the product of the combination of salivary glands in the oral cavity and the components that are derived from the blood by passive diffusion or active transport^7,16^.

Several inflammatory markers in saliva have been associated in response to acute stressors^17–21^. However, a few inflammatory markers have shown correlations between saliva and peripheral blood. Studies that have focused on correlations between salivary and blood-based inflammation were mainly conducted with static, baseline levels (i.e. not in response to stress)^22,23^. Nowadays, within the molecules involved in the inflammatory process, a moderate to strong association has been established only for the non-specific marker of the inflammation, C-reactive protein (CRP), between its serum and salivary levels (r = 0.72, p < 0.001) in non stressful context. Ouellet-Morin and collegues (2011), also identified a dichotomous index of salivary CRP, equivalent to a clinically relevant serum CRP cut-off (3 mg/l), that was associated to known correlates of systemic inflammation^24^. In addition, Riis *et al*. (2014) showed a positive correlation between saliva and blood levels of the IL-1β in healthy adolescent girls after adjusting for age and smoking status^25^.

The salivary inflammatory cytokines could relate to blood-based cytokines in response to stress and/or if examined during a lagged time basis from systemic inflammation. The cytokine responses are not immediate and the impact of mental stress could be delayed for minutes (or even hours) after stress^26,27^.

We previously suggested a significant association between salivary IL-1β and state anger score, supporting that mediators at a local site, as mouth, may indirectly influence emotional processing and changes in motivational state^28^. Likewise, the relationship of salivary IL-6 to psychosocial stress has successful previously demonstrated^21,22^. Interestingly, an important role for IL-18 has been proposed in the stress response. The active and secreted form of IL-1β and IL-18, were generated through the same enzymatic cleavage in the molecular platform named inflammasome. The process is independent by the cellular type that received the stimulus^29,30^. These findings suggest the possibility that like IL-1β, the IL-18 may be a mediator of behavioral change, and that in response to different stimuli, these cytokines have similar induction time and expression trend.

Therefore, saliva has the potential to become a diagnostic sample premier, but there is a need for new standardized methods of assessing inflammation that need to be conducted in environmental contexts, with time and in various conditions. In addition, the use of saliva in stress research could be useful to understand if and how the salivary inflammation markers increase in response to emotional changes.

On the basis of these considerations, we hypothesized that taking saliva samples could represent a reliable surrogate of the plasma sampling under normal or stress conditions. As consequence, the aims of the study were: (i) to examine the plasma *vs*. saliva correlation level of cytokines; (ii) to examine the changes in salivary and plasmatic concentration of the CRP, IL-1β, IL-18, IL-6, and also of the anxiety and anger expression in undergraduate male students on a Resting Day (RD), and in response to a socio-cognitive stressor (Academic Examination, AE).

## Materials and Methods

### Participants

Sixty-one male undergraduate students participated in the study (age: 22–26 years). Written informed consent was obtained from all participants prior to the experiment. The study adheres to the APA Ethical Standards for the treatment of human participants. The study was conducted according to the principles expressed in the Declaration of Helsinki and subsequent revisions and was approved by the Ethics Committee of G. d’Annunzio University, Chieti, Italy.

Volunteers were invited to a preliminary screening session based on a full medical history and examination, the assessment of dietary habits as well as tobacco and alcohol consumption. Habitual smokers were excluded because this factor has already been significantly marked as a strong pro-inflammatory^31^. Subjects who had current infections, allergies, or a present and past history of autoimmune disorders, and those on current medication (including herbal remedies or vitamins) such as anti-inflammatory, antiviral agents or immunosuppressive medication that could directly or indirectly affect the systemic inflammatory state were excluded.

The male participants of the study reported no chronic or acute illness (including periodontal disease), and were in good health in the beginning. The participants were invited not to consume alcohol one week before the sample collection in order to avoid any effects on systemic inflammation^32^. The subjects were told not to undertake excessive physical activity for 48 hours prior to the experiment and to stay away from all kinds of sport for 24 hours before the study. All subjects were asked to stay away from any meals and beverages at least one hour prior to testing time. The average Body Mass Index (BMI) based on 61 participants was within the normal range (M = 22.16, SD = 1.47). Relating of the Psychological assessment, the subjects with high trait anger score (STAXI-2 trait score >75) and/or with high trait anxiety score (STAI-Y trait score >57) were excluded^33,34^.

In order to highlight any low-grade inflammation characterizing healthy subjects, we performed the ELISA test to detect the plasmatic and salivary level of the non-specific marker of inflammation CRP. The students recruited, did not show plasmatic CRP levels >3 mg/L (corresponding to salivary CRP levels >1600 pg/mL) at experimental times^24,35^.

### Procedure

The study was carried out in two days: on RD, a day without an AE, one week before the AE, and the day of the exam (AE). The students attended the first year of the Psychology master degree. The experiment was performed between 12 am to 3 pm in both days because cytokines levels show a circadian rhythm^31,36,37^. On the day of AE, saliva and plasma samples were collected from subjects throughout the study visit (30 min before and 30 min after the examination).

In order to facilitate the taking of blood samples and saliva, the participants were divided into three subgroups. The withdrawals were made taking into account the start and end time of the exam.

### Plasma sampling

Blood samples were collected through veni-puncture on RD, 30 minutes pre- and post-AE. Peripheral blood samples were collected in 4 ml endotoxin-free Heparin tubes (Vacutainer, Becton Dickinson, NJ, USA). Tubes were kept at room temperature and transported to the laboratory for processing within one hour of collection. The plasma was obtained by blood centrifugation as described previously and was kept frozen at −20 °C^38^.

### Saliva sampling

Saliva samples were collected using the Salivette system (Sarstedt Co., Nümbrecht, Germany) during RD and 30 minutes pre- and post-AE. Each of the samples was collected by having the participant place a cotton swab under the tongue for 4 minutes.

To avoid contamination of saliva with blood, participants were instructed not to brush their teeth before the morning saliva samples. Apart from these restrictions, participants were free to follow their normal daily routines on the sampling days. Saliva samples were stored at −80 °C until biochemical analysis. In order to determine blood contamination of saliva samples, a Salivary Blood Contamination Enzyme Immunoassay Kit (Salimetrics, State College, PA, USA) which measures the level of transferrin in saliva samples was used. According to the findings by Schwartz and Granger (2004), the participants with salivary levels of transferrin ≥5 mg/L were excluded^39^.

### ELISA

Salivary and plasma levels of the CRP and pro-inflammatory cytokines IL-1β, IL-18, and IL-6 levels were measured using the commercial kit ELISA (Thermofisher scientific) according to the instructions of the producer (IL-1β ELISA kit, High sensitivity, cat. No. BMS224HS; IL-18 ELISA kit, cat. No. KHC0181; IL-6 ELISA kit, High sensitivity, cat. No. BMS213HS). Plates were scanned using a specialized Charge Coupled Device cooled tool. The integrated density values of the spots of known standards were used to generate a standard curve. Density values for unknown samples were determined using the standard curve for each analysis to calculate the real values in pg/mL. All steps were performed twice and at room temperature. The IL-1β assay sensitivity was equal to 0.05 pg/ml, for the IL-18 it was equal to 12.5 pg/mL, for the IL-6 it was equal to 0.03 pg/ml, and for the CRP was equal to 10 pg/ml. The intra- and inter-assay reproducibility was >90%. Duplicate values that differed from the mean more than 10% were considered suspect and therefore repeated.

We did not reported missing values in salivary evaluation of IL-1β and IL-6 at RD, whereas the use of plasma sample did not permit us to obtain cytokines value from all subjects at the same time.

### Psychological assessment

The anger and the anxiety state level were evaluated in each subject on the RD, and 30 minutes pre and post AE. The anger level of each subject was evaluated by the STAXI-2 questionnaire (State Trait Anger Expression Inventory −2 - Spielberger, 1994)^40^. The STAXI-2 questionnaire is made up of 57 items that measure the experience of anger seen as an emotional state characterized by feelings of different intensities (state of anger), the tendency to perceive a great number of situations as annoying or frustrating (anger trait), and finally as an expression of the same (anger/out, anger/in, control/out, control/in). STAXI-2 is composed of six scales: five sub-scales and an index of anger expressions that can be derived to provide a summarizing measure of the expression as well as the control of anger. The raw scores of every scale were transformed into standardized scores, using tables of conversion from the scores obtained from the Italian Normative Sample^33^.

The anxiety level of each subject was evaluated by the STAI-Y (State Trait Anxiety Inventory - Y - Spielberger *et al*.^34^). The STAI is a self-assessment scale designed to measure both the state and the traits of anxiety. The questionnaire consists of 40 items in which the subject must respond to in terms of intensity (from ‘hardly ever’ to ‘nearly always’). Items are grouped on two scales: Anxiety State, where anxiety is conceived as a particular experience, a feeling of insecurity, helplessness as damage that could lead to worry or to escaping and avoidance; Anxiety Trait, i.e. the tendency to perceive stressful situations as dangerous and threatening and to respond to various situations with a different intensity. The scores obtained from the two scales were interpreted using the reference tables in the Italian Normative Sample^41^. The reference ranges for the scores are as follows: the STAI score >57 high; the STAI score between 40 and 57 normal; STAI score <40 low.

### Statistical analysis

The normal distribution of data was tested by the Skewness and Kurtosis measurement, and analyzed respectively with parametric or non-parametric test. The results were reported separately for each time point: RD, pre- and post-AE. The state of anger, the state of anxiety, and cytokines levels in each experimental time were analyzed by ANOVA for repeated measures followed by the Bonferroni post-hoc test. In accordance with distribution and the scale of the variables, the co-efficient of Pearson correlation (r) was applied to assess the strength of the relationship between the salivary and plasma levels of the CRP, and Δvalue of cytokines (Δcytokine = cytokine post-AE *minus* cytokine pre-AE). Correlations between these immune mediators and Δvalue of anger (ΔR/S) and anxiety (ΔA/S) (Δemotional state score = emotional state score post-AE *minus* emotional state score pre-AE) scores were also evaluated. The ΔA/S and ΔR/S scores were used as a dependent variable in a linear regression analyses, whereas ΔIL-6 and ΔIL-1β were respectively used as independent one.

Statistical analyses were performed using the SPSS 20.0 statistic (SPSS Inc., Chicago, IL, USA) for Windows (IBM). Results are described as means ± SD for each assessment performed in triplicate. All statistical tests were two-tailed and were evaluated at an alpha level of 0.05.

## Results

### Cytokines response

**Figure 1:**
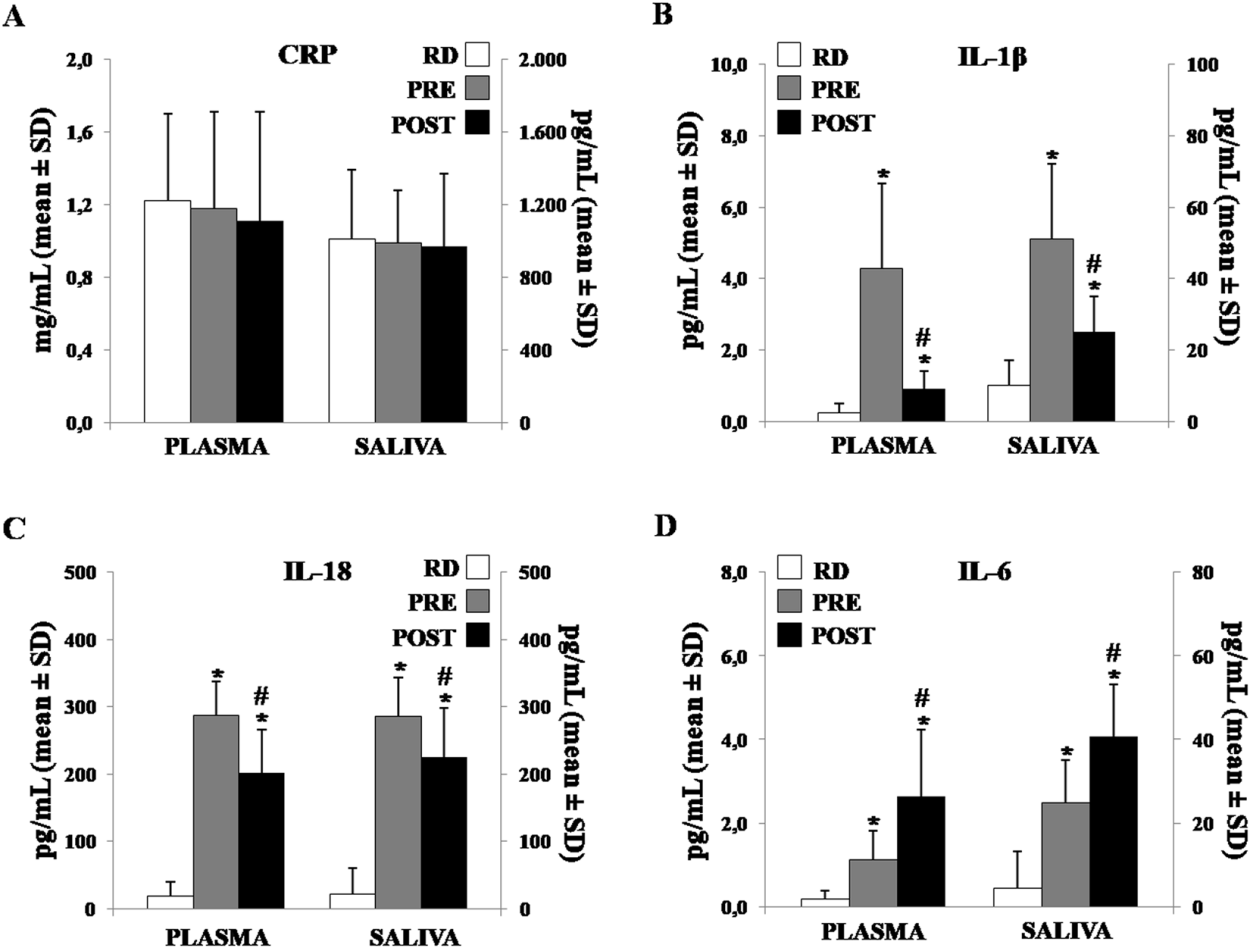
The ELISA measurement of plasmatic and salivary immune markers in male students during the Resting Day (RD) and during the pre- and post-Academic Examination (AE). A significant difference within each experimental time was obtained by ANOVA for repeated measures, considering the evaluation of the within subjects effect. (**A**) The plasmatic and salivary values of the CRP did not show significant differences in plasma nor in saliva. (**B**) Plasmatic and salivary IL-1β concentration significantly changed during the AE. (**C**) Plasmatic and salivary levels of the IL-18 show a similar trend to those observed for the IL-1β. (**D**) Plasmatic and salivary IL-6 levels significantly increased from the RD to the post-AE. *p < 0.01 indicates a significant difference *vs*. previous ascertained time point. ^#^p < 0.01 indicates a significant difference *vs*. RD.

The plasmatic and salivary levels of the CRP, IL-1β, IL-18 and IL-6, were analyzed on the RD, pre and post-AE in each participants in order to determine a possible correlation between plasma and saliva concentrations at the time points considered. As reported in Fig. 1A, data showed that socio-cognitive stress did not significantly modulate the CRP level neither in plasma nor in saliva.

The comparison values between the three experimental times of plasmatic and salivary IL-1β concentrations were reported in Fig. 1B. We found that the IL-1β plasmatic concentration increased significantly pre-AE when compared to the RD. Its levels significantly reduced strictly post-AE (from 4.3 ± 2.7 pg/mL to 0.9 ± 0.5 pg/mL, p < 0.01). At the same time, the salivary concentration of this cytokine showed a similar trend. A peak value in pre-AE (53.4 ± 21.5 pg/mL *vs*. 24.6 ± 9.8 pg/mL, p < 0.01) was reported.

The plasmatic IL-18 value was significantly lower post- when compared to pre-AE (281.7 ± 48.8 pg/mL *vs*. 206.5 ± 35.9 pg/mL, p < 0.05). As showed in Fig. 1C, a similar trend to the pre- *vs*. post-AE for IL-18 salivary levels was found (286.0 ± 45.5 *vs*. 244.3 ± 48.6 pg/mL, p < 0.05).

The plasmatic and salivary levels of the IL-6 show an opposite trend when we considered the pre-AE *vs*. the post-AE, in respect to the other cytokines examined. In detail, the plasma level of the IL-6 detected post-AE was significantly higher than the previous time (2.6 ± 1.4 pg/mL, *vs*. 0.9 ± 0.5 pg/mL, p < 0.05). The same trend in saliva was obtained (42.2 ± 13.1 pg/mL *vs*. 26.2 ± 12.1 pg/mL, p < 0.05) (Fig. 1D).

### Plasma *vs*. saliva correlation

**Figure 2:**
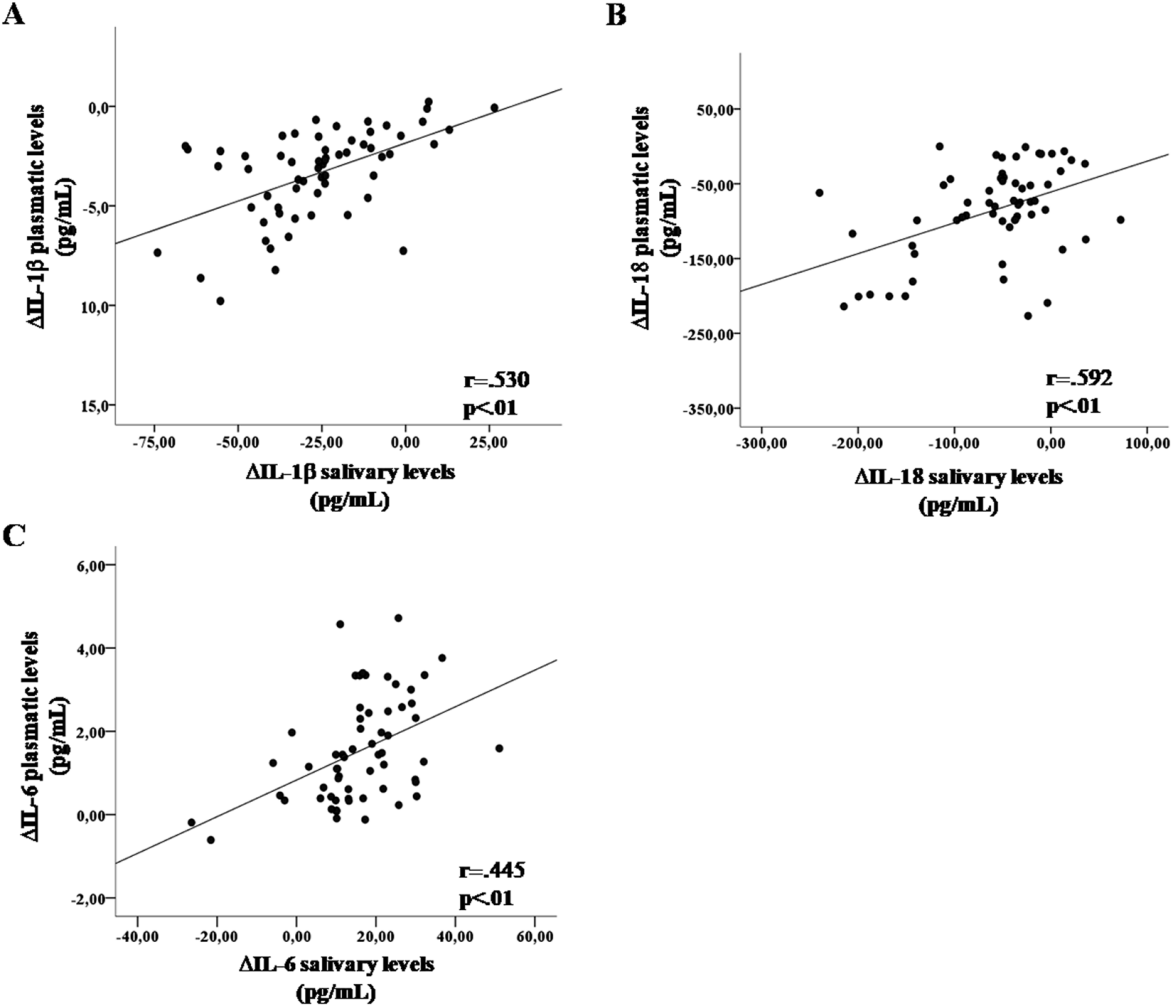
Pearson (r) correlations between plasmatic and salivary levels of ΔIL-1β (**A**), ΔIL-18 (**B**) and ΔIL-6 (**C**) in male students (n = 61). Δcytokine = cytokine post-AE *minus* cytokine pre-AE.

Given a significant modulation recorded over time for the cytokines analyzed, the correlation between plasma and saliva concentration in comparing the variation for each cytokine throughout the AE (Δvalue) was evaluated. Worth noting, is the variation of the concentration in plasma that was significantly and positively associated to the variation in salivary concentration of the IL-1β, IL-18 and IL-6 (Fig. 2A–C). At the RD, no significant correlation was found.

As highlighted above, neither the plasmatic level of the CRP nor its salivary level showed significant differences between the RD and the AE time points. However, both the plasmatic and salivary level were positively associated at each time point (RD, r = 0.403.; pre-AE, r = 0.652; post-AE, r = 0.630; p < 0.01).

### Psychometric scores

**Figure 3:**
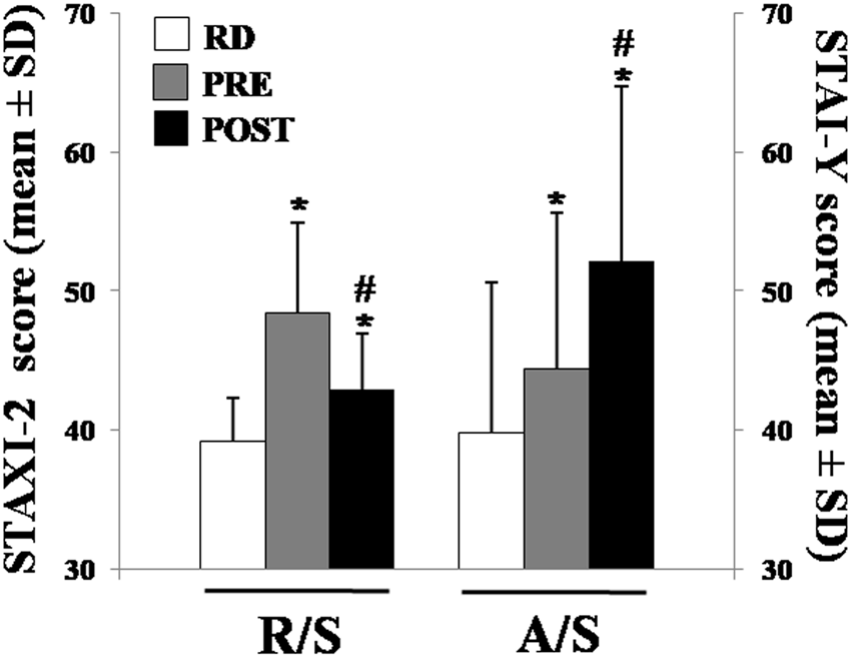
Emotional states variation during Resting Day (RD) and during the pre- and post-Academic Examination (AE) (Socio-Cognitive Stress). The evaluations of the anxiety and of the anger state were performed with a psychometric test. The Rage/State (R/S) score was assessed by the STAXI-2. The Anxiety/State (A/S) score was assessed by the STAI-Y. Significant differences within the R/S score and the A/S score from RD to post-AE were reported. Participants showed a higher score in the R/S score 30 pre-AE when compared to themselves at RD and 30 minutes post-AE. Significant increases in A/S score were reported from the RD to 30 minutes post-AE. Data are expressed as means ± SD (n = 61). *p < 0.01 indicates a significant difference *vs*. previous ascertained time point. ^#^p < 0.01 indicates a significant difference *vs*. RD.

In 61 undergraduate male students, the anger state and the anxiety state were assessed on RD, 30 minutes pre- and post-AE. The anger state was assessed using the STAXI-2, at the same time the anxiety state was directly assessed using the STAI-Y. The results reported in Fig. 3, show that the anger state score was significantly higher on pre-AE when compared to RD and post-AE (48.5 ± 6.4 *vs*. 43.0 ± 4.1, p < 0.01). As to the anxiety state score, an opposite temporal trend was observed. In particular, we reported a significant increase during the examination when compared to RD (41.8 ± 11.3 *vs*. 50.5 ± 12.6, p < 0.01).

### Relations between cytokines response and emotional states

Table 1 shows the bivariate correlations between the plasmatic and salivary level variation across the AE of the pro-inflammatory cytokines ΔIL-1β, ΔIL-18, ΔIL-6 (Δcytokines), as well as the emotional state scores (ΔRS and ΔAS) at the same time. We considered the values obtained 30 minutes pre- and post-AE. On the RD, the variable examined showed no significant correlation. No significant correlation was also found between the plasmatic and the salivary level of CRP as well as the emotional states at each time point. The ΔR/S score correlated positively to the plasma level of ΔIL-1β (r = 0.345, p < 0.01), whereas the ΔA/S score correlated positively to the plasma level of ΔIL-6 (r = 0.423, p < 0.01). None of the variables considered correlated significantly with the plasmatic ΔIL-18.

**Table 1:** Pearson (r) correlation between ΔR/S score, ΔA/S score and immune markers in plasma/saliva (ΔIL-1β, ΔIL-18, ΔIL-6) in healthy undergraduate male students.

	Plasma		Saliva	
	ΔR/S score	ΔA/S score	ΔR/S score	ΔA/S score
ΔIL-1β	0.345**	−0.138	0.426**	0.018
ΔIL-18	0.127	−0.106	0.157	−0.070
ΔIL-6	0.197	0.423**	−0.107	0.341**

The psychometric assessment and biological evaluations were performed 30 minutes pre-socio-cognitive stress (n = 61).

*p < 0.05; **p < 0.01.

As regards to the salivary levels of the analyzed cytokines and their relationship with emotional states throughout the AE, a positive correlation between the ΔR/S score and the ΔIL-1β (r = 0.426, p < 0.01), and the ΔA/S score and the ΔIL-6 (r = 0.341, p < 0.01) was recorded. The ΔIL-18 salivary levels did not correlate to any of the emotional variables.

Linear regression analysis was used to determine whether the temporal variation across AE of the anger and anxiety state score could be explained by the temporal variation of IL-1β and IL-6 levels in plasma and saliva ascertained at the same time.

To determine the relative contribution of each factor, they were introduced independently. The results are shown in Table 2. The ΔIL-1β levels in plasma were significant predictors for the ΔR/S score. The model was significant, F_(1,60)_ = 8.09 (p < 0.001), accounting for 10% of the anger score variance. Interestingly, the ΔIL-1β salivary levels were a better predictor than plasma levels significant accounting for 19% of the ΔR/S score (F_(1,60)_ = 13.32, p < 0.001).

**Table 2:** Regression analysis for Δvalue of IL-1β and IL-6 predicting temporal variation during AE of anger and anxiety score respectively (n = 61).

Dependent Variable	Independent variable	R^2^	R^2^-Adjusted	F_(1,60)_	p-value
ΔR/S score	Plasmatic ΔIL-1β	0.119	0.104	8.09	0.006
	Salivary ΔIL-1β	0.182	0.194	13.32	0.001
ΔA/S score	Plasmatic ΔIL-6	0.179	0.165	13.07	0.001
	Salivary ΔIL-6	0.116	0.101	7.88	0.007

The Δvalue was considered as follow: variable post-AE *minus* variable pre-AE. R/S = Rage State; A/S = Anxiety State.

The ΔA/S score was regressed on ΔIL-6. The analysis showed that during the AE, the variation of plasmatic levels of this cytokine accounted for higher percentage 16% of ΔA/S variance (F_(1,60)_ = 13.07; p < 0.001) when compared to salivary levels (10%). The model was significant (F_(1,60)_ = 7.88, p < 0.01).

## Discussion

This study firstly aimed to investigate the correlation among plasmatic and salivary concentrations of cytokines and CRP in ecological context as the response to a social-cognitive stress (AE). At the same time, we evaluated the relationship among emotional states (i.e. anger and anxiety) and immune markers levels.

Relating to the reliability of the plasma and saliva to measure immune mediators, our results showed a significant plasma-saliva correlation strictly related to the appraisal of socio-cognitive stress, whereas on the RD no significant association was found, except for CRP levels. The outcome suggests that variation in basal salivary cytokine levels in healthy male students, largely reflects the compartmentalized local inflammatory activity of the oral mucosal immune system in response to socio-cognitive stress.

The rise in circulating level of the IL-1β and IL-18 before socio-cognitive stress, reaching a peak value 30 minutes before and then decreased during the examination, could be mainly attributed to an increased bio-synthesis by immune cells^42,43^. For IL-18, these data were in accordance with previous findings showing the induction of the peripheral IL-18 during stress via the activation of the HPA axis after the release of a corticotropic releasing hormone and of the adrenocorticotropic hormone^44^. While the significant rise observed during socio-cognitive stress for the IL-6, could be attributed to increased bio-synthesis by immune cells, and also by the adipose tissue secretion or to the enlargement of the cell pool contributing to circulatory levels^45,46^. On the other hand, the recorded salivary concentrations of IL-6 in the impending of the AE, were more than 20-fold higher than the plasmatic level. This process could be associated with the salivary glands’ activity which translated into several-fold higher levels of the IL-6 in saliva when compared to circulating levels^20^. These differences in the secretion process may differentiate the initiation of the response. Similarly, the IL-1β levels could be dependent by activity of salivary glands and were higher than plasma level across the socio-cognitive stress. The different variations obtained in response to the socio-cognitive-stress, could be due to the different rate of release of IL-1β and IL-6. It is known that TNFα and IL-1β levels rise faster than IL-6 levels. Although other inflammatory cytokines, such as IL-6 and TNFα, are also associated with the responsiveness to stress, IL-1β was the first cytokine to be associated with modulation of neuroendocrine systems, particularly the HPA axis^47–49^.

A positive association was found between the CRP measured in plasma and saliva in pre- and post- socio-cognitive stress, and at the RD. These findings are consistent with results that suggested a significant correlation between plasma and saliva CRP in animal and humans studies^24,50,51^. However, its concentration did not characterize the outcome pattern of the socio-cognitive stress stimulation and cannot be represented as an immune marker of stress.

In the meantime, our findings suggested that anger and anxiety follow an opposite trend in response to the AE. The score assessing anger significantly increased during the examination, while the anxiety state score decreased. In line with this, the subjective appraisal of stressful situations, rather than the stressor itself, gives rise to different emotions. Anger can trigger a cognitive evaluation of some and control, whereas anger could be related to a behavioral approach model. On the other hand, anxiety could be associated with a behavioral inhibition system^52^.

The Δvalue of IL-1β (Δvariable = variable post-AE *minus* variable pre-AE) was positively correlated with the anger score variation when measured in both biological fluids. This positive association is consistent with studies that have highlighted the mediating role of this cytokine in aggressive behavior in animals and humans^28,53^. In general, the association between aggressive behavior and immune activity plays an important adaptive function. It is indeed known that aggressive behavior is critical to maintain a dominant status and to ensure the survival and reproductive success in animals and in humans. On the other hand, aggressive behavior has a high probability of leading to trauma, injuries, and exposure to new diseases. Individuals would benefit from a more effective immune response which in turn promotes the complete recovery of the disease or facilitates the repair of tissue damage in order to respond effectively in case of subsequent re-exposure. Consequently, it is plausible to suppose that the IL-1β play a key role in the initial phase of the response, when the body is predisposed to reacting^43,54,55^.

The concentrations recorded for the IL-18 in plasma and saliva did not correlate with anger and anxiety scores. However, its plasmatic level has been previously correlated with negative emotional ratings in healthy control, and was modulated in pathological conditions, such as schizophrenia, dementia or depression^56–59^. Our study is the first to investigate the role of the IL-18 in physiological conditions, such as stress response, both in plasma and in saliva.

The Δvalue of the anxiety state score was positively correlated with Δvalue of IL-6 in plasma and saliva. These findings acknowledge the association between salivary IL-6 and psychosocial stress, showing that this cytokine was higher in saliva immediately after and after 20 minutes from the completion of a laboratory social test in healthy young adults. Its salivary level also increased in response to prolonged stress in students that were engaged in occupational therapy^20,21,60,61^. In general, cytokines act as both mediators of inflammation and as neuromodulators^62^, and could modulate different emotional states^12^. Circulating plasma cytokines can cross into the Central Nervous System through several pathways. These mediators could act in specific brain sites or remotely by activating critical trigger sites followed by sequential activation of chain reaction to engage distal neural circuits or a combination of the two^62–64^.

Recently, the attention has been focused on the influence of inflammation that is found within peripheral sites, such as the mouth, as well as on behavioral aspects. Some evidence as provided the background for this relationship, including experimental and naturalistic correlation studies. For example, O’Connor *et al*.^5^ showed the positive correlation between Blood Oxygen Level Dependent activity of specific brain areas and salivary IL-1β during a verbal test^5^. Neural pathways involving the trigeminal nerve could directly communicate to local inflammation from the mouth to the brain^65^. Localized expression of inflammatory markers in crevicular fluid (a plasma filtrate emanating from the junction between the oral mucosa and dentine) has also been found in chronic stress (e.g., examination periods) and depression^66,67^. These findings are similar to the well-recognized impact of psychological stress on cellular inflammatory signaling, and on the expression of systemic markers of inflammation^68,69^.

These molecules enter the saliva or via the above mentioned crevicular fluid, or through the transudation of plasma compounds from the oral mucosa, a form of passive transport through osmotic pressure^70^. But they could also originate from increased glandular activity^7,16^. This activity is regulated from the autonomic ganglia in the brainstem which receives input from local sensory nerves as well as from signals emanating from the Central Nervous System. Local activation (e.g. chewing) thus competing with central regulating signals (e.g. affective states, stress).

## Conclusion

Our data supported the emerging role of saliva as a reliable and stress-free tool to evaluate cytokine levels in response to psycho-social stress. In relation to the pro-inflammatory cytokines analyzed, saliva could be considered as a qualitative surrogate for plasma strictly to socio-cognitive stress.

To our knowledge, this is the first study that has analyzed, in an ecological context, the psychobiological response with an integrated approach, focused on the comparison between two of the main tools used to analyze the immune markers. Moreover, a longitudinal design that allows us to draw reliable conclusions regarding the variations of the variables examined across time was adopted^71^. However, it is important to note that there are however limitations to this study. The main one relies on the relatively small sample size. Also, mental and physical illnesses were assessed using a questionnaire and not by a standard medical examination. The results cannot be generalized because the study was composed solely of men. We also did not have access to the academic background of the students, which could have influenced pre-examination stress. However, the biomarkers evaluated in this study could be useful as putative markers of stress response.

In the future, it would be considered appropriate to investigate in ecological context the exact mechanisms by which stress connected to stimuli induces production of cytokines in these biological fluids in healthy subjects.
